# Changes in Physical Fitness during COVID-19 Pandemic Lockdown among Adolescents: A Longitudinal Study

**DOI:** 10.3390/healthcare10020351

**Published:** 2022-02-11

**Authors:** Ting Zhou, Xiangyu Zhai, Na Wu, Sakura Koriyama, Dong Wang, Yuhui Jin, Weifeng Li, Susumu S. Sawada, Xiang Fan

**Affiliations:** 1 College of Physical Education and Health, East China Normal University, Shanghai 200241, China; tzhou@tyxx.ecnu.edu.cn; 2 Graduate School of Sport Sciences, Waseda University, Saitama 359-1192, Japan; xiangyu.zhai@akane.waseda.jp (X.Z.); gnymskr221@akane.waseda.jp (S.K.); wangdonglaser@asagi.waseda.jp (D.W.); 3 Bio-X Institutes, Key Laboratory for the Genetics of Developmental and Neuropsychiatric Disorders, Shanghai Jiao Tong University, Shanghai 200230, China; wuna0308@163.com; 4 Shanghai Key Laboratory of Psychotic Disorders, and Brain Science and Technology Research Center, Shanghai Jiao Tong University, Shanghai 200030, China; 5 Xiamen Foreign Language School Shishi Branch, Xiamen 362700, China; yuhui.jin@hotmail.com (Y.J.); weifeng.liv@hotmail.com (W.L.); 6 Faculty of Sport Sciences, Waseda University, Saitama 359-1192, Japan; 7 Department of Physical Education, Shanghai Jiao Tong University, Shanghai 200240, China; 8 Shanghai Research Center for Physical Fitness and Health of Children and Adolescents, Shanghai University of Sport, Shanghai 200438, China

**Keywords:** COVID-19, lockdown, physical fitness, growth, adolescents

## Abstract

The negative impact of COVID-19 on physical activity has been improved, while the research on changes in physical fitness that may be caused by physical inactivity is still scarce. This study aims to explore the impact of the COVID-19 pandemic lockdown on physical fitness, and the impact of initial physical fitness indicators on their changes during the lockdown in adolescents. A longitudinal study including 265 adolescents aged 14.1 ± 0.4 years old was conducted in China. Physical fitness measurement at baseline and follow-up were respectively measured before (November 2019) and after the lockdown (July 2020). Several physical fitness indicators including aerobic fitness (i.e., 800-m or 1000-m run) and explosive force (i.e., 50-m sprint) deteriorated during the lockdown. Whereas the performances of vital capacity, flexibility (i.e., sit and reach), and muscular strength (i.e., pull-ups) were significantly improved during the lockdown. Furthermore, the reduction in physical fitness for adolescents with higher physical fitness before the lockdown was greater than that for others. These findings may contribute to the development of targeted intervention strategies for physical fitness promotion during the lockdown caused by the public health emergency.

## 1. Introduction

The ongoing global pandemic of Coronavirus disease 2019 (COVID-19) caused by severe acute respiratory syndrome coronavirus 2 (SARS-CoV-2), has resulted in more than 3.38 million deaths worldwide and continues to cause significant morbidity and mortality [1]. To control and stop the spread of COVID-19, many governments established drastic measures including quarantine, lockdown, and community containment. Despite the rigorous government enforcement significantly decreasing the spread of the disease [2], there is a rise in inactivity, therefore increasing the risk of non-communicable diseases [3].

The restrictions during COVID-19 had a dramatic impact on people’s lifestyle behaviors such as diet, sleep, substance use, sedentariness, and physical activity [4,5,6,7]. In particular, the closure of public places and prohibition of social gatherings has reduced the possibility of participating in physical activity outdoors. During the transition between childhood and adulthood, adolescence is an important stage for the development of physical activity, since it can potentially reduce the risks of chronic diseases later in life [8]. However, even in the non-epidemic period, in many countries, 80% or more adolescents do not meet the current World Health Organization physical activity recommendations [9]. A survey of 187 countries showed that physical activity during COVID-19 decreased compared to normal [10], which may have implications on health, such as increased risk of cancer, heart disease, stroke, and diabetes [11,12,13,14]. Compared to these potential risks that are easily ignored, the decline of physical fitness caused by physical inactivity can be evaluated and observed more directly.

Notably, there is a bidirectional relationship between obesity and physical fitness, and fitness predicting obesity was stronger than obesity predicting fitness [15]. Both of them are key factors in the health of adolescents [16,17]. Previous studies indicated that obesity and low physical fitness levels in adolescence were associated with cardiovascular risks and bone health in adulthood [18,19]. In addition to physical health, weight status and physical fitness also play critical roles in mental well-being [20,21]. More specifically, obesity and low physical fitness levels can increase the risks of depression, anxiety, and other common mental health disorders. Moreover, maintaining a normal weight and high aerobic fitness is beneficial to cognitive and brain health [22,23].

To date, most studies emphasized the negative impact of COVID-19 on physical activity, while the research on changes in physical fitness that may be caused by physical inactivity is still scarce. Pinho CS et al. speculated that physical fitness was affected by physical inactivity and sedentary behaviors during COVID-19 [24], which still needs to be further supported by more data. Thus, physical fitness was objectively measured before and after COVID-19 in this study. We hypothesized that (1) body mass index (BMI) has increased and physical fitness has declined after the lockdown; (2) the impact of the lockdown on adolescents with higher physical fitness at baseline was less compared to others, since their initial body function may have long-term effects on their later physical performance [25,26]. Therefore, this study aims to explore the characteristics of the change in physical fitness indicators before and after the COVID-19 pandemic lockdown, and the relationship between these initial physical fitness indicators and their changes during the COVID-19 pandemic lockdown in adolescents.

## 2. Materials and Methods

### 2.1. Procedures and Participants

**Figure 1:**
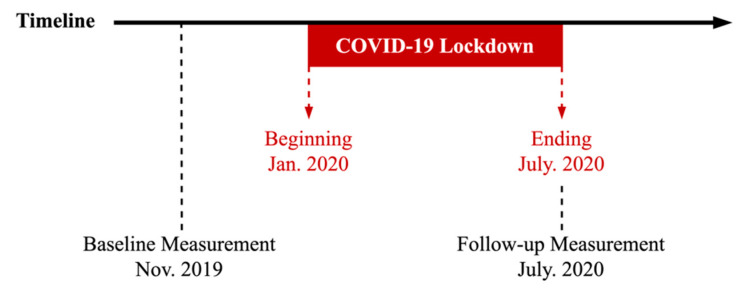
Study design.

A longitudinal study was conducted at a junior high school in Fujian, China. The COVID-19 pandemic lockdown lasted almost 6 months in China (from January to July 2020). During the lockdown, all schools and entertainment places such as malls, fitness centers, and parks were closed; all but essential outings such as medical visits, and food purchases were prohibited by governments. Compulsory physical fitness measurements in November 2019 (baseline measurements) have been conducted every year based on the revised 2014 version of the Chinese National Student Physical Fitness Standard (CNSPFS) [27] as a part of China’s national surveillance. Only adolescents willing to participate in this study were required to undergo the physical fitness measurement and report their birthdates after the COVID-19 pandemic lockdown in July 2020 (follow-up measurements). Both processes of baseline and follow-up measurement were supervised by trained physical education teachers following a standardized survey administration protocol. The study design is presented in Figure 1.

The sample population consisted of adolescents in the second grade of junior high school. A total of 404 adolescents initially participated in the physical fitness measurement at baseline and 308 adolescents completed both physical fitness measurements at baseline and follow-up. Furthermore, 43 adolescents were excluded from our sample due to incomplete measurement data. Ultimately, a total of 265 adolescents (boys, 150; girls, 115) with an average age of 14.1 ± 0.4 years old were included in this study.

### 2.2. Measure

#### 2.2.1. Anthropometry Measures

All participants were required to be barefoot and wear light clothes while measuring body height and weight. Both body height and weight were measured using a portable stadiometer. Body height was measured to the nearest 0.1 cm and body weight was measured to the nearest 0.1 kg. BMI was calculated as the body weight in kilograms divided by the square of the body height in meters (kg/m^2^).

#### 2.2.2. Physical Fitness Measurement

Physical fitness was assessed using eight indicators based on the revised 2014 CNSPFS [27], including vital capacity, 50-m sprint, sit and reach, standing long jump, timed sit-ups, pull-ups, 800-m run, and 1000-m run. Of these indicators, timed sit-ups and 800-m run were assessed only in girls; pull-ups and 1000-m run were assessed only in boys. The rest of the physical fitness indicators were assessed in all adolescents. The specific measurement process of each physical fitness indicator is as follows.

1. Vital capacity; all adolescents were instructed to assess vital capacity using a spirometer in a quiet environment. The test was repeated twice for each adolescent and the better performance from these two tests was recorded.
2. 50-m sprint; all adolescents were instructed to run as fast as possible in a straight line on a 50-m track to assess sprint speed. The test was performed only once for each adolescent, and the time of the 50-m sprint was recorded to the nearest 0.1 s.
3. Sit and reach; all adolescents were instructed to reach forward with their hands as far as possible along a measuring line in a seated position while fully extending both knees and placing feet firmly against vertical support to assess flexibility; the distance was measured to the nearest 0.1 cm. The test was repeated twice for each adolescent and the better performance from these two tests was recorded.
4. Standing long jump; all adolescents were instructed to push off with both feet behind a take-off line and jump forward as far as possible. The distance between the take-off line and the nearest landing point was measured using cm. The test was repeated twice for each adolescent and the better performance from these two tests was recorded.
5. Timed sit-ups; all girls were instructed to perform a timed sit-up test to assess abdominal muscle strength. Laying with knee bent, feet flat on a floor mat, hands placed on the back of the head, and fingers interlocked with each other were required during the test. A complete sit-up movement refers to elevating the trunk until the elbow made contact with thighs and then lowering the trunk until shoulders blades touched the mat. The test was performed only once for each girl, and the number of sit-ups during one minute was recorded.
6. Pull-ups; all boys were instructed to perform a pull-ups test to assess upper-body strength. Grasping an overhead bar by an overhand grip and leaving the ground with both feet were required during the test. A complete pull-up movement refers to pulling the body up using the arms until the chin was above the top of the bar and then lowering the body to the starting position with extended arms. Boys were encouraged to repeat this movement as many times as possible and the number of completed movements was recorded.
7. 800-m and 1000-m run; girls and boys were instructed to run as fast as they could along a track line for 800 m and 1000 m respectively to assess aerobic fitness. Adolescents who were unable to perform the test or had to stop for rest during the test were allowed to walk or jog. The test was performed only once for each adolescent, and the time of the run was recorded to the nearest 0.1 s.

### 2.3. Statistical Analyses

Participant characteristics were described using means and standard deviations. Differences in body height and weight, BMI, and each physical fitness indicator between baseline and follow-up were examined using a paired *t*-test. The change (delta) of physical fitness was calculated by subtracting the data at baseline from the data in follow-up. Pearson correlation coefficient and partial correlation coefficient were used to examine associations between physical fitness indicators at baseline and their changes. Furthermore, all physical fitness indicators were divided into low, moderate, and high groups according to the tertile of their data at baseline, and the differences of their changes among tertile groups were examined using one-way ANOVA and ANCOVA. Multiple comparisons were examined using Fisher’s least significant difference (LSD). The acceptable threshold of statistical significance was specified as 0.05 (two-tailed). All statistical analyses were conducted using the R program (4.0.3 version).

## 3. Results

A total of 265 adolescents (age: 14.1 ± 0.4 years old) were included in this study, with a higher number of boys (*n* = 150; 56.6%) than that of girls (*n* = 115; 43.4%). The participant characteristics are presented in Table 1. In girls, only body height and weight increased significantly after the lockdown, but there was no significant difference in BMI. Furthermore, the performance of vital capacity and sit and reach of girls in follow-up were significantly better than that at baseline. While the performance of the 50-m sprint and the 800-m run decreased significantly after the lockdown in girls. In boys, body height and weight, and BMI all increased significantly after the lockdown. Moreover, the performance of vital capacity, sit and reach, and pull-ups in follow-up of boys were significantly better than that at baseline. While the performance of the 50-m sprint and the 1000-m run declined significantly after the lockdown in boys.

The results of the correlation coefficient between physical fitness at baseline and its changes are shown in Table 2. Except sit and reach of girls and the vital capacity and 50-m sprint of boys, all other physical fitness indicators at baseline were significantly correlated with their corresponding changes. In girls, the correlations of vital capacity, 50-m sprint, and 800-m run are weak, with a range from −0.303 to −0.207 and a range from −0.305 to −0.208 after controlling for the effects of age; standing long jump and timed sit-ups at baseline were moderately associated with their changes (R = −0.533 to −0.434; R^a^ = −0.534 to −0.431) during the lockdown. In boys, the correlations of standing long jump, sit and reach, pull-ups, and 1000-m run are weak, with a range from −0.360 to −0.241 and a range from −0.348 to −0.242 after controlling for the effects of age.

**Figure 2:**
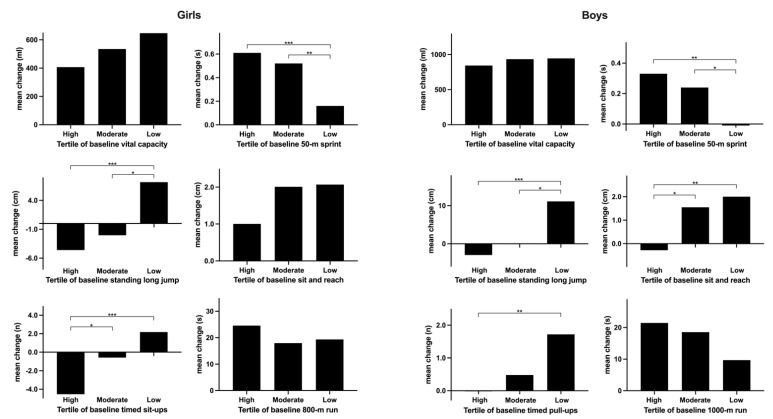
Differences of mean change in physical fitness among tertile groups in girls and boys. * *p* < 0.05, ** *p* < 0.01, *** *p* < 0.001.

As shown in Table 3, differences in mean changes in physical fitness among baseline tertile groups were examined. Multiple comparisons between tertile groups in girls and boys are respectively presented in Figure 2. Girls who were initially in the high or moderate tertile group had a greater decline in several physical fitness indicators including 50-m sprint, standing long jump, and timed sit-ups than those in the low tertile group. Similarly, boys with high or moderate physical fitness indicators in the 50-m sprint, standing long jump, sit and reach, and pull-ups at baseline also had a greater decrease than those with moderate or low physical fitness performance.

## 4. Discussion

This longitudinal study evaluated (1) the changes in BMI and different physical fitness indicators including vital capacity, flexibility, muscular fitness, and aerobic fitness before and after the COVID-19 pandemic lockdown; (2) the relationship between the initial physical fitness and its changes during the COVID-19 pandemic lockdown in adolescents. The results of this study could be beneficial for developing a targeted intervention strategy for physical fitness promotion during the lockdown caused by the public health emergency.

Despite that, physical activity levels were observed to decrease in many countries during the COVID-19 pandemic lockdown [10], contrary to expected, not all physical fitness indicators were negatively affected by the lockdown. In this study, pulmonary function (i.e., vital capacity) and flexibility (i.e., sit and reach) at follow-up were significantly higher than that at baseline in both girls and boys. The improvement of these physical fitness indicators is plausible since pulmonary function and flexibility usually increase during adolescence due to physical growth with age [28,29]. In addition, the increase in muscular strength was only observed in boys, not in girls. Previous evidence proved the increase of muscle strength does not depend on gender until the age of 14 years [30]. We speculated that the increase in muscle strength resulting from physiological development is greater in boys than in girls, possibly because energy metabolism and sex hormones at puberty stimulate the development of muscle bulk in boys, but fat accumulation in girls [31]. The age of the participants in our study was around 14 years, a stage of rapid muscle development for boys. Therefore, we considered that physical inactivity caused by the COVID-19 lockdown may delay the growth of muscular strength to some extent. However, the positive impact that physical growth has on muscular strength is greater than the negative impact that lockdown had in boys in our study.

However, Sunda et al. found a more negative trend in muscular fitness among boys aged 16.25 ± 0.55 years at baseline [32]. This inconsistency with our study may be because their fiber size had stabilized at that time. A longitudinal study showed that peak fiber size plateaued at the age of 16 [33]. It means the positive impacts of physiological development on muscular strength at 16 years could not offset the negative impacts caused by COVID-19.

Despite the drastic impact of COVID-19 globally, the specific effects on human physical fitness with different age groups and conditions in various countries have not been fully explored. The physical fitness of adolescents aged 14 years should increase as changes in body shape and composition occur naturally with puberty [34]. However, in this study, we found that explosive force and aerobic fitness decreased after the COVID-19 pandemic lockdown. Hence, although the impact of physiological development on fitness changes could not be adjusted, we considered that the lockdown might negatively affect their health by comparing the results with previous studies [29]. This may provide some information for future research covering a broad age range as the change of physical fitness may be the result of the combined effect of age and COVID-19 [35].

Unlike increases in the above physical fitness indicators including vital capacity, flexibility, and muscle strength, both aerobic fitness (i.e., 800-m or 1000-m run) and explosive force (i.e., 50-m sprint) significantly decreased in all adolescents after the lockdown in this study. While the decrease in maximal oxygen intake was observed only in Spanish girls [36]. This inconsistency may be the result of different lengths of the lockdown. The lockdown in China lasting almost 6 months (from January to July 2020) is much longer than that of 6 weeks in Spain. At present, there are no published studies regarding explosive force to compare. Interestingly, our findings showed that not all physical fitness indicators declined during the COVID-19 pandemic lockdown, which differs slightly from our initial hypothesis. This suggests that the lockdown may delay the growth of different physical fitness indicators to a different degree. One possible explanation for this is that limited exercise space in the home environment may result in fewer exercise patterns to choose from such as stretching and resistance training. Beyond that, the explanation for the underlying physiological mechanism needs to be further explored in the future.

Another important finding of this study is that physical fitness at baseline was negatively associated with their changes, which was in contrast to our initial hypothesis. In other words, the impact of the lockdown was greater for adolescents with higher physical fitness compared to others. Actually, this unexpected finding is reasonable. Based on the previous studies showing a positive relationship between healthy lifestyles and physical fitness level [37], we assumed that adolescents with higher physical fitness at baseline may have a healthier lifestyle under normal circumstances compared to others. Due to the sudden lockdown, people’s normal lifestyles were disrupted, leading to restrictions on outdoor activities, an increase in sedentary time, unbalanced diets, and other passive unhealthy behaviors [38,39]. It is reported that reduced physical activity and unlimited or unbalanced food intake impair our body, especially for adolescents who are in a period of rapid development [40,41]. Therefore, we speculated that the COVID-19 lockdown particularly negatively affected adolescents with healthy lifestyles initially, which might indirectly result in their huge reductions in physical fitness. Based on the above, it is suggested that the impact of the lockdown on lifestyle may be reflected by the changes in physical fitness.

The main strength of this study is the use of longitudinal design and objective and comprehensive measurement for physical fitness, including vital capacity, flexibility, explosive force, muscular strength, and aerobic fitness. To the best of our knowledge, this is the first study showing the differing impact of the lockdown on multiple physical fitness indicators. Additionally, the results suggested that adolescents with higher physical fitness levels were more likely to be affected by the lockdown.

Some limitations of this study include: first, physical fitness at baseline was measured two months before the COVID-19 pandemic lockdown. It would be ideal if this physical fitness measurement was conducted close to the date the COVID-19 pandemic lockdown began (the end of January 2020), although it is hard to do so since the outbreak and lockdown of the COVID-19 pandemic occurred without any warning; second, the impact of physiological development on results cannot be adjusted, since there were no data on fitness changes without COVID-19 lockdown in this study; thirdly, the results of this study may not be generalizable to all Chinese adolescents since the sample was recruited from the same junior high school in China; lastly, other potential variables such as physical activity level during the lockdown and socioeconomic features have not been considered, which may have an impact on the results of this study.

## 5. Conclusions

Findings from this longitudinal study indicate that not all physical fitness indicators deteriorated during the COVID-19 pandemic lockdown in adolescents. Only reductions in aerobic fitness and explosive force were observed. In addition, the reduction in physical fitness for adolescents with higher physical fitness before the COVID-19 pandemic lockdown was greater than that for others. In summary, the decline in fitness performance may be due to the lack of physical activity caused by the restrictions on outings and the limitation of space for exercising.

Although months of lockdown have effectively controlled the spread of the COVID-19 pandemic, it also indirectly led to the decline in fitness. Therefore, developing targeted guidelines and policies for adolescents to promote health during the lockdown caused by public health emergencies is necessary and crucial. For instance, managers can open fitness centers appropriately while ensuring social distancing under the guidance of public health departments; educators can develop online physical education courses.

Furthermore, applicable and specific programs of exercise should also be developed for limited space for movement. According to the results of our study, it is suggested that there should be an emphasis on explosive force and aerobic fitness. Adolescents with high fitness should pay more attention to their health status, as they were more vulnerable to poor health by the lockdown.

## Figures and Tables

**Table 1:** Participant’s characteristics at baseline and follow-up.

	Variables	Baseline		Follow-Up		*p* Value
		Mean	SD	Mean	SD	
Girls *n* = 115	Age	14.2	0.3	14.8	0.3	
	Body height (cm)	159.6	5.7	161.5	5.3	<0.001
	Body weight (kg)	50.6	8.4	52.2	8.7	<0.001
	BMI (kg/m^2^)	19.8	2.8	20.0	2.9	0.12
	Vital capacity (mL)	2512.5	498.8	3042.2	610.1	<0.001
	50-m sprint (s)	8.9	0.6	9.4	0.7	<0.001
	Standing long jump (cm)	154.7	20.7	154.7	19.8	0.98
	Sit and reach (cm)	9.2	6.5	10.9	6.9	<0.001
	Timed sit-ups (*n*)	36.2	10.6	35.2	9.1	0.16
	800-m run (s)	226.9	21.9	247.6	25.2	<0.001
Boys *n* = 150	Age	14.1	0.4	14.8	0.4	
	Body height (cm)	164.6	8.0	169.3	7.3	<0.001
	Body weight (kg)	53.9	12.6	58.9	13.0	<0.001
	BMI (kg/m^2^)	19.8	3.9	20.5	3.9	<0.001
	Vital capacity (mL)	3037.2	706.2	3944.7	832.3	<0.001
	50-m sprint (s)	8.0	0.7	8.2	0.8	<0.001
	Standing long jump (cm)	185.2	26.5	188.1	26.7	0.08
	Sit and reach (cm)	2.0	7.0	3.0	7.3	0.003
	Pull-ups (*n*)	2.0	3.0	2.8	3.6	0.002
	1000-m run (s)	262.0	34.3	279.3	38.6	<0.001

Note: SD, standard deviation; BMI, body mass index.

**Table 2:** The correlation coefficient between baseline and change in physical fitness.

	Variable	R (Baseline vs. Delta)	*p* Value	R ^a^	*p* Value ^a^
Girls	Vital capacity (mL)	−0.207	0.03	−0.208	0.03
	50-m sprint (s)	−0.303	0.001	−0.305	0.001
	Standing long jump (cm)	−0.434	<0.001	−0.431	<0.001
	Sit and reach (cm)	−0.159	0.09	−0.160	0.09
	Timed sit-ups	−0.533	<0.001	−0.534	<0.001
	800-m run (s)	−0.243	0.009	−0.248	0.008
Boys	Vital capacity (mL)	−0.107	0.19	−0.113	0.23
	50-m sprint (s)	−0.138	0.09	−0.139	0.14
	Standing long jump (cm)	−0.360	<0.001	−0.348	<0.001
	Sit and reach (cm)	−0.241	0.003	−0.242	0.010
	Pull-ups (*n*)	−0.296	<0.001	−0.296	0.001
	1000-m run (s)	−0.249	0.002	−0.249	0.007

Note: SD, standard deviation; BMI, body mass index; ^a^ adjusted for age.

**Table 3:** Differences of mean change in physical fitness among baseline tertile groups.

	Variable	High		Moderate		Low		*p* Value	*p* Value ^a^
		Mean	SD	Mean	SD	Mean	SD		
Girls	Vital capacity (mL)	406.95	390.38	534.67	480.71	647.11	510.36	0.08	0.08
	50-m sprint (s)	0.61	0.58	0.52	0.43	0.16	0.54	0.001	0.001
	Standing long jump (cm)	−4.60	18.26	−2.03	12.85	7.16	11.91	0.002	0.002
	Sit and reach (cm)	1.00	2.50	2.01	3.06	2.07	4.65	0.31	0.31
	Timed sit-ups (*n*)	−4.54	4.75	−0.59	5.39	2.18	9.71	<0.001	<0.001
	800-m run (s)	24.56	22.39	17.91	12.48	19.32	19.79	0.28	0.29
Boys	Vital capacity (mL)	842.96	527.43	934.08	516.95	945.38	527.19	0.56	0.52
	50-m sprint (s)	0.33	0.41	0.24	0.70	−0.01	0.55	0.007	0.007
	Standing long jump (cm)	−2.96	13.75	0.04	18.71	11.13	22.15	<0.001	0.001
	Sit and reach (cm)	−0.28	3.66	1.55	3.11	2.00	4.95	0.010	0.007
	Pull-ups (*n*)	−0.02	3.32	0.48	1.08	1.72	3.07	0.005	0.005
	1000-m run (s)	22.41	31.49	17.24	24.07	12.22	28.10	0.19	0.23

Note: SD, standard deviation; BMI, body mass index; ^a^ adjusted for age.

## Data Availability

Data are not publicly available due to protect participants’ privacy, but are available from the corresponding authors on reasonable request.
